# Comments on “Reporting quality of randomized controlled trial abstracts in the seven highest-ranking anesthesiology journals”

**DOI:** 10.1186/s13063-019-3857-7

**Published:** 2019-11-19

**Authors:** Rohan Kumar Ochani, Asim Shaikh, Naser Yamani

**Affiliations:** 1 Department of Internal Medicine, Dow University of Health Sciences, Baba-e-Urdu Road, Saddar, Karachi, Pakistan; 2 Department of Internal Medicine, Rush University Medical Center, Chicago, IL USA

**Keywords:** Reporting quality, CONSORT for abstracts, randomized controlled trials

## Abstract

Randomized controlled trials are considered the gold standard in assessing treatment regimens, and since abstracts may be the only part of a paper that a physician reads, accurate reporting of data in abstracts is essential. The CONSORT checklist for abstracts was designed to standardize data reporting; however, for papers submitted to anesthesiology journals, the level of adherence to the CONSORT checklist for abstracts is unknown. Therefore, we commend Janackovic and Puljak for their efforts in determining the adherence of reports of trials in the highest-impact anesthesiology journals between 2014 and 2016. The results of their study are extremely important; however, we believe that that study had some methodological limitations, which we discuss in this manuscript.

Dear Editor,

The importance of adhering to the Consolidated Standards of Reporting Trials (CONSORT) checklist when reporting randomized controlled trials cannot be overstated, as the results of a trial can strongly influence clinical practice [1], especially for abstracts, since busy clinicians often rely solely on the abstract. Hence, we commend Janackovic and Puljak for their efforts in determining the adherence of reports of trials to the CONSORT checklist for abstracts in the highest impact anesthesiology journals between 2014 and 2016 [2]. The results of their study are extremely important; however, we believe that that study has some methodological limitations.

Firstly, that study calculates an overall total adherence score for all trials. All items in the checklist were scored as “yes,” “no,” or “unclear.” Hence, that study clearly assigns an equal weight to each item on the CONSORT checklist. We believe that giving each item an equal value and scoring them identically is not the best approach, as evidently some items should carry much more importance, such as randomization, blinding, and reporting of the primary outcome compared to giving the contact details of the authors [3]. Furthermore, the total adherence score is heavily influenced by a very few items that have extreme results. In that study, “interventions,” “objective,” “outcome,” and “conclusions” all had scores of over 90% and in contrast, “source of funding” had a score of only 0.2%. We suspect that these values had a profound impact on the total adherence score.

Secondly, the study also states “two authors independently screened bibliographic results.” An inter-rater reliability test, such as Cohen’s kappa, would have been of great benefit here. Multiple individuals collecting similar types of data often come to different conclusions. Moreover, variables that are subject to inter-rater errors are common throughout the clinical literature [4]. Therefore, while resolving discrepancies via discussion may have produced a consensus, conducting an inter-rater reliability test would have identified discrepancies and which variables were susceptible to errors. That study does not indicate the level of agreement achieved for these crucial differences.

Finally, the study compares the total adherence scores obtained for each journal and states which had the highest and lowest scores. Note that journals can have very different reporting criteria and policies for certain items [5]. Some journals insist that certain items are reported in the full text as opposed to the abstract, and vice versa. Moreover, there can be discrepancies between an abstract and its corresponding full text [6]. Therefore, comparing journals based on their total adherence scores may be misguided. Perhaps, comparing individual checklist items between journals, especially important items such as allocation concealment, would be more effective at highlighting significant inadequacies concerning adherence to CONSORT.

## Data Availability

Not applicable.
